# The Sulfate-Reducing Microbial Communities and Meta-Analysis of Their Occurrence during Diseases of Small–Large Intestine Axis

**DOI:** 10.3390/jcm8101656

**Published:** 2019-10-11

**Authors:** Ivan Kushkevych, Oľga Leščanová, Dani Dordević, Simona Jančíková, Jan Hošek, Monika Vítězová, Leona Buňková, Lorenzo Drago

**Affiliations:** 1 Department of Experimental Biology, Faculty of Science, Masaryk University, Kamenice 753/5, 62500 Brno, Czech Republic; Lescanova@mail.muni.cz (O.L.); vitezovam@sci.muni.cz (M.V.); 2 Department of Plant Origin Foodstuffs Hygiene and Technology, Faculty of Veterinary Hygiene and Ecology, University of Veterinary and Pharmaceutical Sciences, 61242 Brno, Czech Republic; dordevicdan@vfu.cz (D.D.); janickovasim@vfu.cz (S.J.); 3 Regional Centre of Advanced Technologies and Materials, Faculty of Science, Palacky University in Olomouc, 78371 Olomouc, Czech Republic; jan.hosekj@upol.cz; 4 The Department of Environmental Protection Engineering, Faculty of Technology, Tomas Bata University in Zlín, 76001 Zlín, Czech Republic; bunkoval@utb.cz; 5 Department of Biomedical Sciences for Health, University of Milan, 20122 Milan, Italy; lorenzo.drago@unimi.it

**Keywords:** bowel disease, colitis, small–large intestine axis, sulfate reduction, hydrogen sulfide

## Abstract

Sulfate-reducing bacteria (SRB) are often isolated from animals and people with ulcerative colitis and can be involved in the IBD development in the gut–intestine axis. The background of the research consisted of obtaining mixed cultures of SRB communities from healthy mice and mice with colitis, finding variation in the distribution of their morphology, to determine pH and temperature range tolerance and their possible production of hydrogen sulfide in the small–large intestinal environment. The methods: Microscopic techniques, biochemical, microbiological, and biophysical methods, and statistical processing of the results were used. The results: Variation in the distribution of sulfate-reducing microbial communities were detected. Mixed cultures from mice with ulcerative colitis had 1.39 times higher production of H_2_S in comparison with samples from healthy mice. The species of *Desulfovibrio* genus play an important role in diseases of the small–large intestine axis. Meta-analysis was also used for the observation about an SRB occurrence in healthy and not healthy individuals and the same as their metabolic processes. Conclusions: This finding is important for its possible correlation with inflammation of the intestine, where the present of SRB in high concentration plays a major part. It can be a good possible indicator of the occurrence of IBD.

## 1. Introduction

Sulfate-reducing bacteria (SRB) represent probably a trigger for the occurrence of inflammatory bowel diseases (IBD) since studies are connecting their presence with these diseases, especially their metabolic end product H_2_S in the gut [1,2]. Other ailments (including rheumatic diseases and with ankylosing spondylitis) occur also in their presence [3]. SRB use sulfate as an electron acceptor in the process of dissimilatory sulfate reduction. The final product of this process is hydrogen sulfide [4]. Constant microorganism cultivation is happening in the large intestine since certain undigested food remains in it. [1,2]. Around 200 g of digestive material is found in the large intestine of an adult human [2,3,5,6]. These bacteria are in the fermentation process can cleave complex organic compounds and form molecular hydrogen, different acids (acetic and lactic), same as other compounds. Lactic acid bacteria fermentative properties are directly responsible for the production of lactate [4]. Other groups of microorganisms can also use lactate and acetate, serving as electron donors and carbon sources [7,8,9,10,11,12]. The important role of human physiological processes is their capability to absorb sulfate and develop amino acids out of it (cysteine and methionine). The amount of the sulfate present in the intestine is related to human diet [13,14,15,16], meaning that it is highly influenced by individual’s eating habits. The importance of daily sulfate intake can be overseen by the fact that staple food commodities represent high sulfate sources (>10 µmol/g) [13].

Although, sulfate amounts that are not used in amino acid synthesis represent good conditions for SRB [1,4,17,18,19,20,21]. SRB needs electron acceptor (sulfate serves this purpose) and they form hydrogen sulfide as their final product [22,23,24,25,26,27]. An exogenic electron donor, including lactate can be also used and oxidized to acetate [18,28]. The dominant SRB in the intestine of humans is *Desulfovibrio* genus [5,22,28]. The studies are emphasized connections between the presence of SRB in the intestines and the prevalence of ailments, such as cholecystitis, brain abscesses, and abdominal cavity ulcerative enterocolitis. Sulfate-reducing bacteria are not the only ones that produce H_2_S in the intestinal content. Numerous bacterial groups convert cysteine to H_2_S, pyruvate, and ammonia by cysteine desulfhydrase activity [2,3,4,12].

Though connections have been found, it is still not clear how these processes are affecting the prevalence of certain ailments. Meta-analysis is used widely in medical research, as in natural science. It is included in systematic reviews as a rigorous method for mapping the evidence gained by many authors. The meta-analysis should provide unbiased overviews of multiple results and should assess evidence quality and synthesize it. The first step of a systematic review is the research question that is deconstructed by sample consideration, the second step is intervention and then come outcome and comparator. The outcome of the meta-analysis depends on the study field, but in many cases, quantitative results are used [29].

The aim of the research was to compare a variation in the morphological distribution of sulfate-reducing microbial communities from healthy mice and mice with colitis, their production of hydrogen sulfide, and to study the occurrence of these bacterial populations during diseases of the small–large intestine axis.

## 2. Experimental Section

### 2.1. Manipulation with Animals

Male C57Bl/6 mice (20 g ± 2 g) were obtained from the Animal Breeding Facility of Masaryk University (Brno, Czech Republic). They were kept under standard conditions (22 ± 2 °C, 50 ± 10% relative humidity) and alternating 12 h light/dark cycles. The animals had access to a standard diet and drinking water ad libitum. Manipulations with the animals were carried out according to the bioethical rules as per the principles of the “European Convention for the Protection of Vertebrate Animals Used for Experimental and Other Scientific Purposes” adopted in Strasbourg in 1986. The study was also approved by the “Commission for the Protection of Animals against Cruelty” and the Ethics Committee of the University of Veterinary and Pharmaceutical Sciences in Brno, Czech Republic. In total, six animals in two groups (4 + 2 animals in the first and second group, respectively) were randomly separated and used in this experiment. In the dextran sulfate sodium (DSS) group (*n* = 4), colitis was induced by administering 5% (*w*/*v*) DSS (MP Biomedicals, Illkirch-Graffenstaden, France, MW 36,000–50,000 Da) in drinking water for 7 days. The mice in the intact group (*n* = 2) received drinking water only. On the last day of the experiment, the animals were killed by decapitation under isoflurane anesthesia. The isolated distal colonic segments were selected for the analysis of the qualitative and quantitative composition of intestinal microflora of both groups of the animals.

### 2.2. Bacterial Mixed Cultures

The material used for the study consisted out of mixed sulfate-reducing bacteria cultures that were isolated from feces of healthy and with ulcerative colitis mice. After the autopsy, the samples were placed in the tubes. The bacteria were studied as mixed cultures because the aim of the study was not the purification of SRB. Mixed cultures were kept at the Laboratory of Anaerobic Microorganisms of the Department of Experimental Biology at Masaryk University (Brno, Czech Republic).

### 2.3. Cultivation of SRB Cultures

SRB cultures were cultivated according to Kovac and Kushkevych (2017) [30] and Postgate (1984) in a modified Postgate C medium [23]. Mohr’s salt (ammonium iron sulfate hexahydrate, Sigma-Aldrich, Prague, Czech Republic) was used as a simple growth detection. Ferrous salt forms reacted with sulfide produced by SRB (dark black precipitate of FeS) and indicated the presence of SRB (the presence of dissimilatory sulfate reduction). Due to the method, it was possible to optically determine the presence of metabolic activity qualitatively and quantitatively.

The cultures were kept in medium with Mohr’s salt and without is since color changes are not desirable for spectrophotometric and turbidimetric methods. In cultures kept in medium without Mohr’s salt, the SRB can be detected by the sharp smell of hydrogen sulfide same as by optical turbidity. The medium was sterilized (pH 7.5–7.7, E_h_ = −100 mV). Redox potential was adjusted by Na_2_S (Sigma-Aldrich, Prague, Czech Republic) and ascorbic acid (Sigma-Aldrich, Prague, Czech Republic). The anoxic atmosphere was ensured by the nitrogen gas addition, inhibiting oxygen from the air to diffuse into the medium. The oxygen proof layer was secured by the addition of paraffin (Sigma-Aldrich, Prague, Czech Republic) drops to each cultivation tube. The strains were able to grow 10 days under these conditions. 

The long storage (up to one month) conditions for cultures were provided by Postgate B medium with the addition of Mohr’s salt. In this medium there is always tending of bacteria to descend to the bottom of the tube due to the presence of the precipitate. Bacteria usually stick to the walls of the tube when is used modified Postgate C medium.

### 2.4. Description of Morphology

Microscope Olympus BX50 (lympus, Japan) was used for the observation of cells.

Phase-contrast microscopy is a technique that allows images of transparent specimens (living cells). The advantage of this technique is the possibility to do the measuring without cell killing since cells can be monitored with real-time motility. The bacterial suspension (a drop) was placed on a glass slide. The slide (cover glass added to the top of bacterial suspension) was analyzed immediately after immersion and with 100× objective.

The Gram staining method provides observation of gram-positive and gram-negative bacteria by differential staining with the use of crystal violet-iodine complex and a safranin counterstain. Gram-positive bacteria appear purple after treatment with alcohol while gram-negative bacteria appear pink. After drying samples were microscopically observed, including oil immersion 100× objective.

Capsule staining. Acidic and basic stains cannot be used for bacterial capsules. Therefore, the best way to visualize them is to stain the background using an acidic dye (e.g., nigrosine, Congo red) and to stain the cell itself using a basic stain (e.g., crystal violet, safranin, methylene blue). One drop of Congo red dye was mixed with one drop of bacterial suspension on a glass slide. After spreading throughout the slide and letting dry, it was immersed in hydrochloric acid (4 mol/L) and after a few seconds, it was let dry again. Subsequently, methylene blue dye was added on the slide and it was let standing for three minutes. After three minutes, the slide was washed with deionized water, dried, and observed with immersion oil and 100× objective. The cells were stained blue and their capsules remained white and visible on a dark background.

DAPI (4′,6-diamidino-2-phenylindole) staining is a fluorescent dye, binding by preference to the AT-rich regions of DNA [31]. Microorganisms with thick cell walls can be stained with DAPI after permeabilization of the cell wall by ethanol. For this type of microscopy, using a 48-hour old culture was found most suitable. A 48-h-old cell suspension of a volume 25 µL to 100 µL was diluted in several ml of MiliQ deionized water and washed by vacuum filtration. After washing, the filtration paper with cells was let dry. Consequently, 20 µL of DAPI stain (Sigma-Aldrich, Prague, Czech Republic) was applied and the filtration paper with cells was kept in the dark in a refrigerator for 10 min. After that, the filtration paper was washed in water, ethanol, and water, respectively, and let dry. Next, it was put on a glass slide with immersion oil applied both under and over the filtration paper with cells, and the slide was observed in a microscope, using WU filter (Sigma-Aldrich, Prague, Czech Republic) and 100× objective.

### 2.5. pH Tolerance and Temperature Range Test

As measured before, the optimal pH for the cultivation of intestinal SRB is from 7 to 8 [15]. The measuring was done by performing a simple pH test. The modified Postgate C medium was prepared by adjusting various pH values, performed by adding drops of sodium hydroxide (aqueous solution) and hydrochloric acid (aqueous solution), respectively. CyberScan 510 pH-meter (PreSens, Regensburg, Germany) was used to measure the exact pH values (pH ranged from 4 to 12). Media were heated to 37 °C in Wasserman tubes inoculums (obtained from healthy and not healthy mice) of cultures. Paraffin oil (500 µL) was added on the top of the medium to provide an oxygen-proof layer. The optical density of the suspension was measured at 430 nm using spectrophotometer Spectronics Genesys 5 (Thermo Fisher Scientific, Prague, Czech Republic). Blank samples were media without inoculum. Optical density was measured after 24 h of cultivation again. Bacteria were added in Eppendorf tubes and placed in thermostats (1-CUBE, Havlickuv Brod, Czech Republic) set at 5, 25, 35, 45, 50, and 60 °C. Optical density was measured at 430 nm using Spectronic Genesys 5, after 72 h of cultivation. 

### 2.6. Production of Hydrogen Sulfide

**Figure 1:**
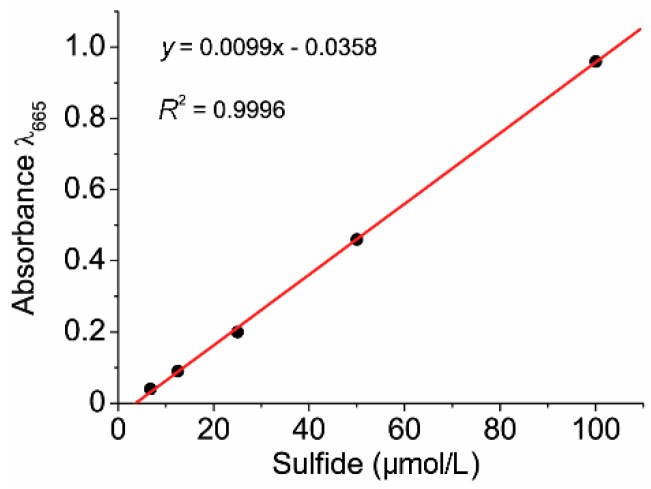
The calibration used for the determination of sulfide concentrations.

Spectrophotometrical methylene blue method was used for measuring the presence of hydrogen sulfide in solution [32]. The bacterial suspension (1 mL) was pipetted to 5 mL of aqueous zinc acetate (5 g/L). 2 mL of p-aminodimethylaniline (Sigma-Aldrich, Prague, Czech Republic) solution (0.75 g/L in 2 M sulfuric acid) was added immediately and the solution was let stand at room temperature for 5 min. 0.5 mL of ferric chloride (FeCl_3_) (12 g/L in 0.015 M sulfuric acid) solution was consequently added. The solution was centrifuged at 2200 RPM (10 °C for 5 min). After centrifuging, the samples lost the original light pink color and had a blue color. The absorbance was measured at 665 nm by Spectronic Genesys 5 spectrophotometer. The procedure for blank sample preparation included preparation that a clear cultivation medium was added in step 1. The concentrations used for calibration solutions ranged from 6 µmol/L to 100 µmol/L (Figure 1).

### 2.7. Statistical Analysis

Using the experimental data, the basic statistical parameters (M—mean, m—standard error, M ± m) were calculated. The accurate approximation was when *p* ≤ 0.0533 [33]. Statistical analysis was done by SPSS 20 statistical software (IBM Corporation, Armonk, NY, USA). Plots were built by software package Origin 7.0 (Northampton, MA, USA).

Meta-analysis consisted of studies found on the WEB OF KNOWLEDGE database. The database found 38 studies, from the year 1945 to 2019.considering sulfate-reducing bacteria. Only six studies were included in the meta-analysis since other studies did not satisfy the specific hypothesis of the study. The Review Manager Software (Cochrane, Brno, Czech-Republic) (number 5.3 developed by Cochrane Collaboration) was used. In the included studies the data consisted of the number of participants with the positive occurrence of the SRB bacteria in the group of healthy people and people with ulcerative colitis. In other studies, the data consisted of the mean, standard deviation and the number of the measurements. Heterogeneity was expressed by the *I^2^* test, where the higher *I^2^* represented a higher heterogeneity. 

## 3. Results

**Figure 2:**
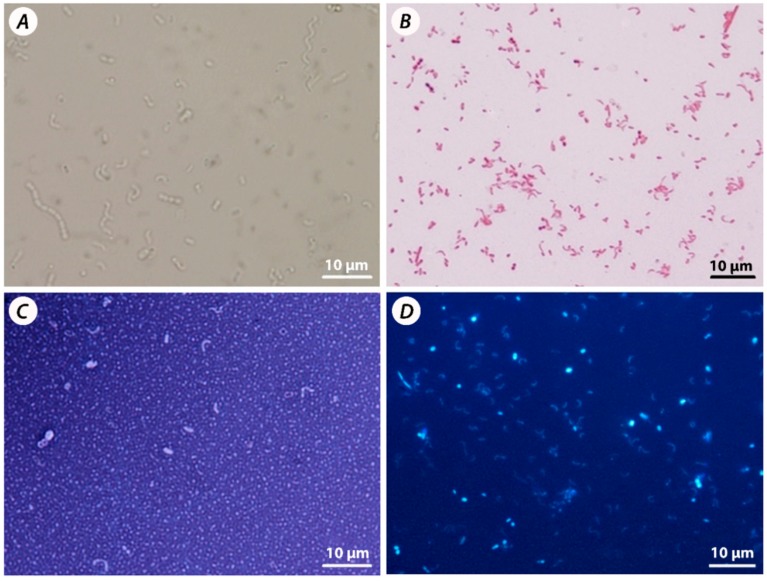
Sulfate-reducing bacteria (SRB) mixed culture: native slide (**A**), Gram staining (**B**), capsule staining (**C**), DAPI staining (**D**).

The vibrio shape was a dominant shape of the cells, as expected. Though they are very small and thin that makes them very often hard to be observed. These cells were marked as *Desulfovibrio* sp. Due to their characteristic shape, gram negativity and flagellar motility (Figure 2). Very abundant were also cells, oval form. Chain and cluster shaped had cocci that were larger than vibrios, same as some rod shape cells were observed too. Rods have almost similar characteristics as cocci. Not abundantly spirilloid forms of bacteria were present too. They had long shape and were very thin, curved multiple times (maximum twelve curves) (Figure 2A). They had long, polar flagella that are responsible for rapid movement. Gram-negative bacteria only were not only present in SRB cultures isolated from rodents (Figure 2B).

*Desulfotomaculum* is rod-shaped (stained Gram-positive) (representing non-SRB genera in the gut) can be seen in Figure 2C since it has a short rod oval shape. According to the previous microscopic technique, cocci can be encapsulated or not. More often encapsulated cocci are present in pairs. The formation of capsules occurs probably due to a non-favorable environment, such as high hydrogen sulfide concentrations due to sulfate-reducing bacteria presence. It is important to stress out that capsule formation is not defined as SRB characteristic. DAPI (4′,6-diamidino-2-phenylindole) staining is compliant with the observations made by the previous technique (Figure 2D). The most abundant was vibrio cell-shape. SRB present in the gut isolate was probably *Desulfovibrio* sp., according to literature data that is describing them as the most frequently isolated species in the intestinal inflammation environment. Cocci were confirmed by DAPI staining since they are significantly brighter and larger than other cells. The findings that DAPI cultures bind to DNA molecules indicate that some oval-shaped have more DNA than others, meaning that they are unrelated to each other. Different sizes of cocci, gained by previous techniques, is supporting this interpretation. These cells were found in multiple isolates because thin rods of exceeding length were found by DAPI staining. These cells represent a common microbiome in the intestines that are capable to survive in conditions designed for SRB cultivation. 

**Figure 3:**
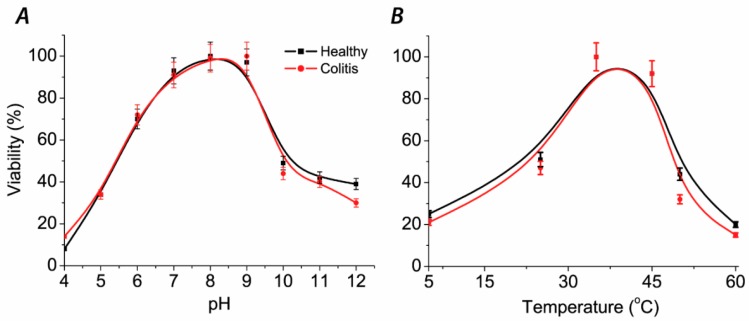
Various pH (**A**) and temperature (**B**) influence on relative viability of SRB cultures.

The fastest bacterial growth and viability, measured spectrophotometrically OD_430_ (Figure 3), was detected after 24 h of cultivation at 37 °C and pH from 8.0 to 9.0. A significant drop in viability was observed at pH 10. The absence of black precipitate was observed in tubes with Mohr’s salt and pH > 10 (Figure 3A). This result is indicating a threshold limit pH ≥ 10 both for sulfate-reducers and other (contaminating) species. The values did not reach zero value but were stabilized at around 30–40% of maximum bacterial growth. It means that bacteria were capable to survive and divide at this pH, reaching an optical density of 0.3. Black precipitate occurred at all pH values, meaning that bacteria can survive a longer time period before starting to metabolize and produce hydrogen sulfide. The changing of color in the tubes at pH 11 and 12 occurred due to basic conditions. It means that the measured values of optical density can be explained by the extreme pH effect. 

After 72 h of cultivation bacterial growth of all samples was observed. SRB cultures can grow at various ranges of temperature conditions, not only at 37 °C, though the fastest growth occurred at temperature ranges from 37 °C to 45 °C. Another observation was that cells survived for three days at 50 °C and died on the temperatures higher than 60 °C and at the temperature of 5 °C (no bacterial growth, no hydrogen sulfide production, black precipitate not occurred and low OD_430_ values were measured. The growth was slow at a temperature of 25 °C. The relative viability values of SRB are shown in Figure 3B.

**Figure 4:**
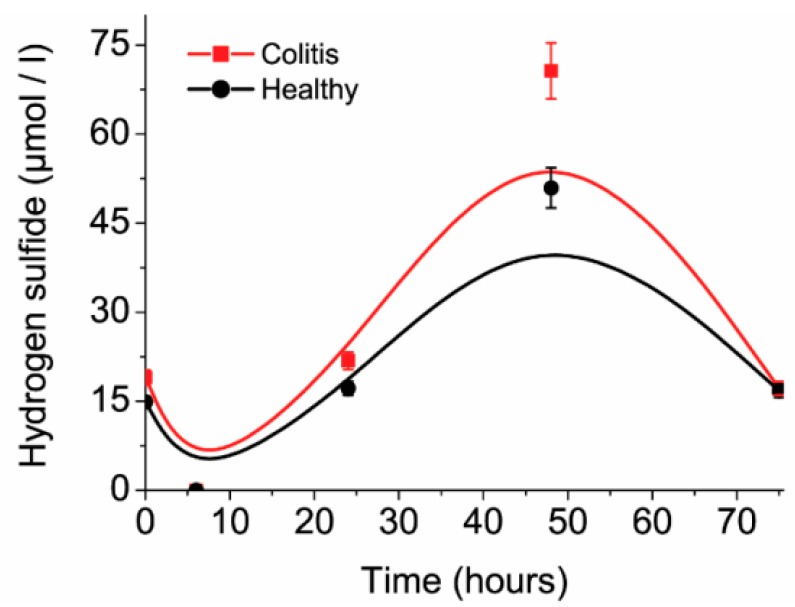
Amount of hydrogen sulfide in cultivation medium in 72 h.

The concentrations of H_2_S in time change according to cell number, same as their metabolic activity rate. The maximum measured hydrogen sulfide concentrations were measured after 48 h of cultivation (Figure 4). After 48 h of cultivation H_2_S concentrations decreased due to the decrease in relative substrate concentration in the medium, though H_2_S can clear out from the medium. H_2_S is present in a soluble form in the medium and can be released as the gaseous phase (the presence of a bubble under the lid, accompanied by hydrogen sulfide sharp smell) into the environment. Consequently, sulfide concentrations dropped at the beginning of the cultivation. After six hours of cultivation, soluble sulfide was eliminated into gaseous phase and it was a point where the lowest H_2_S levels were detected. Mixed cultures from mice with ulcerative colitis had 1.39 times higher production of H_2_S in comparison with samples from healthy mice. The maximal difference was 20 µmol/L after 48 h of cultivation.

**Figure 5:**
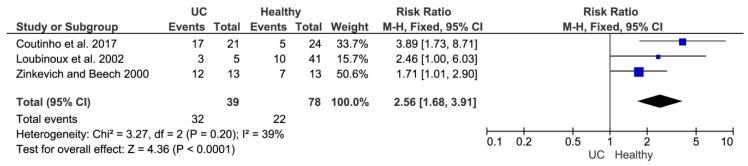
The occurrence of SRB in a group of healthy people and patients with UC.

It should be noted that sulfate-reducing microbial communities from healthy mice and mice with colitis were used only as of the model objects for confirmation of morphology distribution and hydrogen sulfide production in different groups of animals (healthy and with ulcerative colitis). Another part of the study consisted of a literature data overview that was conducted by meta-analysis. This method was used for comparing SRB prevalence in healthy individuals and people with developed inflammatory bowel disease. The occurrence of SRB in a group of healthy people and patients with ulcerative colitis (UC) was studied (Figure 5). The location of the square on the right side means that not healthy people are more likely to experience SRB. A significant difference in the occurrence of SRB in healthy people can be observed in the first study [34]. The other two studies [5,35] already touch the zero effect line at a 95% confidence interval, so there is no significant difference. The diamond can then be seen on the right side. Summary of the studies found that SRB is less common in healthy people than in people with UC.

**Figure 6:**
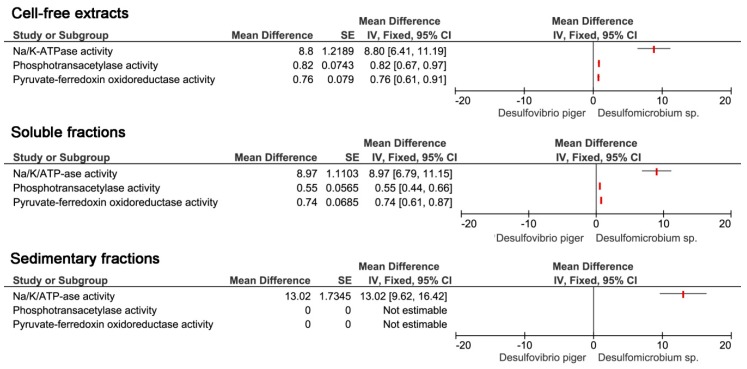
Enzyme activity in *Desulfovibrio* and *Desulfomicrobium*.

The production of hydrogen sulfide occurs in the process of dissimilatory sulfate reduction, where tree main enzymes are involved. Since the species of *Desulfovibrio* genus were dominant among SRB in both mice and people with ulcerative colitis, the activity of the enzymes involved in the processes of sulfate reduction in *Desulfovibrio* and other intestinal SRB *Desulfomicrobium* was compared (Figure 6). In the case of enzyme activity in cell-free extracts, it was found that in all cases it had the lower enzymatic activity of *Desulfomicrobium* sp. phosphotransacetylase and pyruvate-ferredoxin activity was more or less the same in *Desulfovibrio* bacteria. Thus, it can be argued that the activity of Na^+^/K^+^ ATPase is the highest of the investigated enzymes in the cell-free extracts of *Desulfovibrio*. Similar results were observed in soluble fractions. The activity of Na^+^/K^+^ ATPase is highest in *Desulfovibrio* than *Desulfomicrobium* in all enzymes examined. In the case of sediment fractions, higher Na^+^/K^+^ ATPase activity was again found in *Desulfovibrio* bacteria and no activity was observed in both *Desulfovibrio* and *Desulfomicrobium* in the other investigated enzymes, phosphotransacetylase, and pyruvate-ferredoxin oxidoreductase.

Thus, the contribution of sulfate-reducing microbial communities, especially of the *Desulfovibrio* genus, in both groups of healthy people and patients with UC and enzymatic activities of bacterial cells is based on a meta-analysis is obvious. Though, the number of studies is certainly not enough for a stronger conclusion.

## 4. Discussion

Important factors that influence the intestinal environment are sulfate consumption, sulfide production, lactate consumption and acetate accumulation [7,8,9,10]. Very often Desulfovibrio genus is present in the intestines and feces of people and animals with inflammatory bowel disease, meaning that this genus plays an important role in the development and occurrence of this ailment. Sulfate is used as a terminal electron acceptor by these bacteria, the same as organic compounds are used as electron donors in their metabolism [6,7]. Leading us to the conclusion that sulfate in food commodities (some bread, soya flour, dried fruits, brassicas, and sausages, as well as some beers, ciders, and wines) play an important role in the development of bowel disease [13].

The principal component analysis showed that the *Desulfovibrio* strains from individuals with colitis grouped in one cluster by biomass accumulation and sulfide production, while the strains from healthy individuals formed another cluster that included the same parameters. A negative correlation (Pearson correlations, *p* < 0.01) was found between sulfate and lactate consumption. Biomass accumulation and hydrogen sulfide showed lower linear regression (*R*^2^). The kinetic parameters, biomass accumulation, and sulfide production have an important role in bowel inflammation, including ulcerative colitis. Acetate produced by SRB probably has a synergy interaction with H_2_S since sulfate consumption and lactate oxidation represent minor factors in bowel disease [16].

Optimum growing conditions for the bacteria were provided by the study. The intensive growth of *D. piger* Vib-7 was observed in the presence of higher electron acceptor and donor concentrations. Consequently, the intensive accumulation of sulfide and acetate occurs too. According to previous studies and literature data, these conditions are the probable cause of ulcerative colitis, leading to bowel cancer. Hydrogen sulfide negatively affects intestinal mucosa, epithelial cells, the growth of colonocytes [4,14,15,16,17,18,36,37,38,39], causes phagocytosis, causes the death of intestinal bacteria [4,12,24], and induces hyperproliferation and metabolic abnormalities of epithelial cells [12]. The presence of SRB and high level of metabolites are also connected with colon inflammation [4,6,38]. Hydrogen sulfide concentrations are regulating the integrity of colonocytes [37,38,39]. In the samples of individuals with ulcerative colitis was also found that SRB sulfide production is higher [5,6]. According to another study dealing with the SRB metabolic process was found that the strains isolated from people with colitis shifted to the right side of the Y-axis by biomass accumulation, sulfate consumption, lactate oxidation, same as hydrogen sulfide and acetate production, in comparison with the strains isolated from healthy individuals. The percentages were differences observed in shifting to the right side of the Y-axis: biomass accumulation 26%, sulfate consumption 1.5%, and sulfide production 5% [14]. The intestinal microbiota is a complex system, interactions occur between clostridia, methanogens, lactic acid bacteria, etc. Though, SRB plays a central role in the development of IBD, including ulcerative colitis [1,2,3,11]. Lactic acid bacteria, methanogens, and many other intestinal microorganisms can be inhibited by hydrogen sulfide produced by SRB [2].

Preservatives added to food often contain sulfur oxides, sulfate polysaccharides (mucin), chondroitin sulfate, carrageenan, and other food commodities represent the source of sulfate and lead to evaluated sulfate intake in the daily diet that leads to increase of hydrogen sulfide concentrations produced by SRB. The western diet contains over 16.6 mmol sulfate/day [13] and the feces of about 50% of healthy individuals contain SRB (*Desulfovibrio*: up to 92%) [1,5,24]. On the other hand, the concentrations of hydrogen sulfide are toxic not only for the intestinal environment but also for their producers. The concentrations higher than 6 mM stop the growth of *Desulfovibrio*, but metabolic activity was not 100% inhibited (the results supported by cross-correlation and principal component analysis). 5 mM concentrations of H_2_S resulted in two times and eight times longer lag phase and generation time, respectively [18]. It should be noted that clostridia can also produce hydrogen sulfide, but in smaller quantities and can be interacted with SRB [40] Terminal oxidative processes in the large intestine of humans can be also included in the activities of SRB. The connections between SRB presence and activity in the intestine and occurrence of ulcerative colitis were also found in animal studies where SRB isolated from mice with UC produced 1.14 times (higher hydrogen sulfide production rate can damage aggressively intestinal mucosa) more sulfide ions than SRB isolates from healthy mice [6].

It is of crucial importance that all issues concerning H_2_S metabolic processes and its influence on the gastrointestinal environment are well studied and tested. Since it has been observed in animal studies that H_2_S-releasing agents can be seen as promising therapeutic agents for many indications [41]. H_2_S is confirmed to represent an important signaling factor for cardiovascular and nervous systems statute [42]. The way how cecal musoca protects itself from the toxical effects of H_2_S is the conversion to thiosulfate. Consequently, these metabolic pathways play an important role in the occurrence of ulcerative colitis [43]. The importance of similar studies can be seen through the fact that mechanisms leading to Chron’s disease still remain unclear [44].

According to meta-analysis, SRB occurs more often in patients with UC. The finding can be explained by the fact that counts of SRB are lower (though still detectable) in healthy individuals. Oppositely, in patients with developed inflammatory bowel disease, the production of H_2_S reaches toxic levels and also destroyed its producers (sulfate-reducing bacteria) [15]. 

## 5. Conclusions

Sulfate-reducing bacteria are present in various environments and they make a high impact on animal and human health since their presence is a possible contributing factor in the development of inflammatory bowel diseases. Their morphology (vibrio, spiral, rods, and cocci) and diversity are highly influenced by environmental conditions including temperature, pH, oxygen presence and substrate availability. Unique in nature is anaerobic sulfate-reducing bacteria metabolism in which hydrogen sulfide is produced in the process of electron acceptors (mainly sulfate ions) reduction (the process of dissimilatory sulfate reduction). The study clearly showed that mixed SRB cultures obtained from healthy and with ulcerative mice were equally polymorphic (the most often vibrio and coccus shape occurred). Though, the production of hydrogen sulfide differs significantly among isolated cultures. It was observed that isolates from not healthy mice produced higher hydrogen sulfide amounts. This observation is emphasizing correlations between intestine inflammation occurrence and hydrogen sulfide concentrations. The meta-analysis confirmed these correlations. Presently, it is still not fully understood the occurrence processes of inflammatory bowel diseases, including ulcerative colitis. Though, the study is emphasizing one more time that the occurrence of SRB in the samples with developed IBD is pointing out the importance of issues concerning sulfate-reducing bacteria.
